# Analytical and Structural Studies for the Investigation of Oxidative Stress in Guanine Oligonucleotides

**DOI:** 10.3390/ijms21144981

**Published:** 2020-07-15

**Authors:** Györgyi Ferenc, Zoltán Váradi, Zoltán Kupihár, Gábor Paragi, Lajos Kovács

**Affiliations:** 1 Nucleic Acid Synthesis Laboratory, Biological Research Centre, Temesvári krt. 62, H-6726 Szeged, Hungary; ferenc.gyorgyi@brc.hu; 2 Nucleic Acids Laboratory, Department of Medicinal Chemistry, University of Szeged, Dóm tér 8, H-6720 Szeged, Hungary; varadi8008@gmail.com (Z.V.); kupihar.zoltan@med.u-szeged.hu (Z.K.); 3 MTA-SZTE Biomimetic Systems Research Group, Dóm tér 8, 6720 Szeged, Hungary; 4 Institute of Physics, University of Pécs, Ifjúság útja 6, 7624 Pécs, Hungary

**Keywords:** oxidative stress, epigenetic, reactive oxygen species, reactive nitrogen species, guanine, quadruplex, 8-oxo-7,8-dihydro-2′-deoxyguanosine, analytical methods, structural investigations, computational studies

## Abstract

DNA damage plays a decisive role in epigenetic effects. The detection and analysis of DNA damages, like the most common change of guanine (G) to 8-oxo-7,8-dihydroguanine (OG), is a key factor in cancer research. It is especially true for G quadruplex structure (GQ), which is one of the best-known examples of a non-canonical DNA arrangement. In the present work, we provided an overview on analytical methods in connection with the detection of OG in oligonucleotides with GQ-forming capacity. Focusing on the last five years, novel electrochemical tools, like dedicated electrodes, were overviewed, as well as different optical methods (fluorometric assays, resonance light scattering or UV radiation) along with hyphenated detection and structural analysis methods (CD, NMR, melting temperature analysis and nanopore detection) were also applied for OG detection. Additionally, GQ-related computational simulations were also summarized. All these results emphasize that OG detection and the analysis of the effect of its presence in higher ordered structures like GQ is still a state-of-the-art research line with continuously increasing interest.

## 1. Introduction

**Scheme 1:**
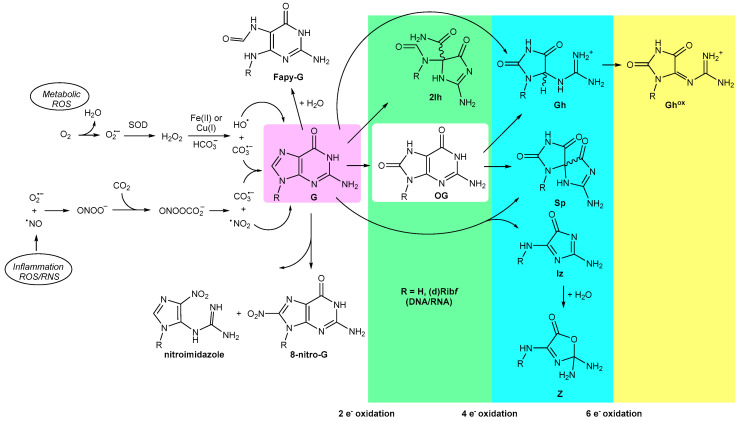
The oxidative transformation of guanine oligonucleotide derivatives upon reaction with reactive oxygen species (ROS) and reactive nitrogen species (RNS). To have a better overview, not all radical intermediates have been included (adapted from References [5,18,19,20]).

The presence of an oxidative environment in which aerobic organisms strive constitutes a constant threat. Coping with this condition required the elaboration of distinct mechanisms to deal with the reactive oxygen species (ROS) involved in this process. In our current understanding, ROS have been utilized in upregulation and as messenger molecules during inflammation. The process of this gene regulation is possible due to the special feature of guanine (G) residues in nucleic acids, namely, their easier oxidation compared to other nucleobases and their ability to form tetrads (G4) and quadruplexes (GQs). The oxidation of guanines paired in the quadruplex is twice as fast as oxidation of the same sequence in a duplex context [1,2]. The primary product of G from ROS exposure is 8-oxo-7,8-dihydroguanine (OG (R = H, Scheme 1), for the sake of simplicity, the nucleobase term OG is used throughout this review unless the oxidized DNA nucleosides are specifically referred to). It is estimated that ≤100,000 OGs are formed per cell daily, and it is the most frequently measured biomarker of ROS-induced DNA oxidation to date [3]. OG is a target for base excision repair initiated by the enzyme OG-glycosylase 1 (OGG1). Traditionally, the formation of OG is considered as a biomarker of a mutagenetic event [4] but it could be epigenetic by functioning as a transcriptional regulator [5,6] or, in general, as a tool to improve the genome plasticity to redox changes [7]. The formation of OG is either beneficial or detrimental depending on the gene product that is formed from the OG-containing promoter [5]. OG may alter gene expression and its transcriptional impact is dependent on the coding versus template strand of OG occurrence, and it can temporarily stop or even completely abolish mRNA synthesis. The role of guanine lesions is the complex process of epigenetic regulation that has been reviewed recently by several authors [1,5,7,8,9,10,11,12,13,14,15,16,17]. Pertinent to this multifaceted role is the analytical detection, quantification and structural studies of the facilitated formation of OG in G-rich oligonucleotide sequences, in particular, G-quadruplexes and their hydrolysis products (nucleosides, nucleobases), which is the subject of the present review.

## 2. Scope and Limitations

The formation of OG is a two-electron oxidation event followed by further oxidative and hydrolytic transformations, resulting in additional products (Scheme 1). The frequency of OG formation is ca. 1 in 10^6^ [18], while that of the four-electron (spiroiminodihydantoin (Sp), 5-guanidinohydantoin (Gh), and imidazolone (Iz)) and six-electron oxidation (5-guanidino- dehydrohydantoin (Gh^ox^)) and hydrolysis products (2,6-diamino-4-hydroxy-5-formamido- pyrimidine (Fapy-G), oxazolone (Z)) is much lower [18,19], therefore our treatment is limited mostly to the analytical and structural studies related to OG (the formation of OG dominates under aerobic conditions, while the yield of Fapy-G increases under anaerobic conditions [18]). The temporal frame of our review encompasses papers from the last five years with occasional reference to earlier papers when justified.

## 3. Analytical Detection and Quantitation Methods

### 3.1. The Concentration of ROS, Reactive Nitrogen Species (RNS) and the Characteristic Processes Leading to Their Formation

The typical concentration of oxygen, the source of metabolic ROS, ranges from ∼20 to 100 μM O_2_ depending on the tissue type [5]. While the partially reduced superoxide radical ion (O_2_^•−^) forms in only 0.1–2% yield, it is still generated in significant amounts. Superoxide has a short cellular half-life (∼1 μs) and it is the subject of disproportionation catalyzed by superoxide dismutase (SOD) to furnish hydrogen peroxide (H_2_O_2_), with a half-life of ∼10 μs. The steady-state concentration of hydrogen peroxide is ∼200 nM and can increase many folds under high metabolic demand or stress. In general, the concentration of ROS is generally high, and its elevated load can overburden the natural defense systems, leading to inflammation. Hydrogen peroxide is a signaling agent for immune cell activation and vascular remodeling. A side reaction of H_2_O_2_ is the Fenton reaction to yield the aggressive one-electron oxidant hydroxyl radical (HO^•^; E_red_ = 2.31 V versus normal hydrogen electrode (NHE)) when H_2_O_2_ reacts with iron(II) [21] or copper(I) ions [22]. In the presence of physiological bicarbonate concentration (∼25 mM), the Fenton reaction yields primarily carbonate radical anion (CO_3_^•−^) [23]. Reactive nitrogen species (RNS) also contribute to inflammatory responses (there are ca. 80 known DNA defects that can be attributed to ROS or RNS) [11,20]. Among others, peroxynitrite ion (ONOO^−^), the intracellular concentration of which lies in the nanomolar range [20], reacts with cellular CO_2_ to yield short-lived nitrosoperoxycarbonate anion (ONOOCO_2_^−^). The ion ONOOCO_2_^−^ affords, in a homolytic degradation, the one-electron oxidants carbonate radical anion (CO_3_^•−^; E_red_ = 1.59 V versus NHE) and nitrogen dioxide radical (^•^NO_2_; E_red_ = 1.04 V versus NHE) in ca. 70% yield (the steady-state levels of ^•^NO_2_ in activated macrophages have been estimated to be in the picomolar range) [20]. The ion ONOOCO_2_^−^ preferentially causes oxidation and nitration of G in DNA and probably RNA, it is likely one of the major oxidizing species in vivo due to millimolar concentrations of CO_2_ in tissues, and the large reaction rate constants of CO_2_ and ONOO^−^ ion [20]. G is the most susceptible to oxidative modification among the canonical nucleobases (E_red_ = 1.29 V versus NHE) [20,24]. It was also demonstrated that the catalytic one-electron oxidation kinetics in poly-GC sequences is particularly rapid [25]. The carbonate radical anion specifically oxidizes G to OG in high yields. Nitrogen dioxide radical is incapable of oxidizing G to OG but it readily oxidizes OG further (0.74 V versus NHE) as an OG is at least 1000-fold more reactive than the parent G toward further oxidation [20]. In addition, unselective one-electron oxidation of duplex DNA can yield an electron hole that can migrate through thousands of base pairs, yielding oxidation at the most sensitive G-rich sites. This feature confers a sacrificial “cathodic protection” character to G tracts to prevent deterioration of other nucleobases [24].

### 3.2. Electrochemical Methods

The electrochemical methods belong to the most sensitive analytical methods, and therefore their privileged role in the detection of mutagenic or epigenetic OG formation is well motivated owing to its low yet significant occurrence in vivo. Over the years, several sophisticated procedures have been developed to monitor and quantify samples containing the nucleobase OG, its precursor nucleoside 8-oxo-7,8-dihydro-2′-deoxyguanosine (o^8^dGuo, in the literature frequently abbreviated as 8-OHdG as well) or even its presence in ONs or GQs.

#### 3.2.1. Development of New Electrodes

##### Electrodes Coated with Reduced Graphene Oxide Nanocomposites

**Figure 1:**
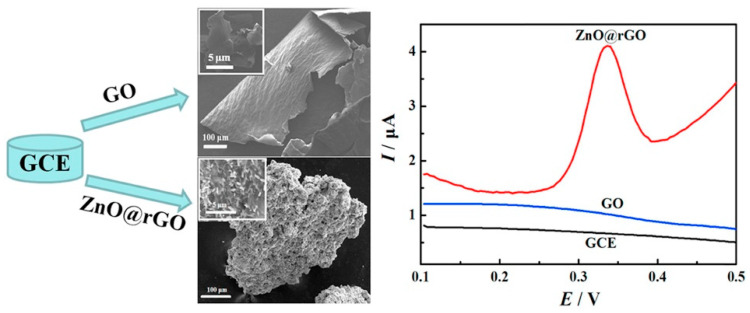
ZnO nanoparticles (ZnO@rGO) for the detection of o^8^dGuo. Reprinted with permission from Reference [26], © 2018, Elsevier.

Hao et al. [26] have prepared a nanocomposite using reduced graphene oxide (rGO) and ZnO nanoparticles (ZnO@rGO) via an in situ reduction of graphene oxide (GO) with Zn powder and coated glassy carbon electrode (GCE) (Figure 1). The ensuing system (ZnO@rGO/GCE), characterized by scanning electron microscopy (SEM) and transmission electron microscopy (TEM), gave significantly enhanced oxidation signals of o^8^dGuo, compared with unmodified GCE and GO-modified GCE (GO/GCE), as verified by cyclic voltammetry (CV). With the enzyme uricase, the interference of uric acid (UA) was effectively eliminated and accurate sensing of o^8^dGuo was attained. The linear range of 5 to 5000 nM for the detection of o^8^dGuo using ZnO@rGO/GCE has been achieved using differential pulse voltammetry (DPV). The limit of detection (LOD) was 1.25 nM.

rGO has also been combined with another metal oxide, dysprosium oxide (Dy_2_O_3_) nanoparticles, for the determination of o^8^dGuo in human blood and urine samples by Manavalan et al. [27]. Dy_2_O_3_@rGO was synthesized by a microwave-assisted synthetic route to improve the moderate electrical conductivity; furthermore, the electrocatalytic ability was synergically improved. The electrochemical and interfacial properties were examined by electrochemical impedance spectroscopy (EIS). Under optimum conditions, the electrocatalytic performances of Dy_2_O_3_@rGO-modified electrode and control electrodes were analyzed by CV. The Dy_2_O_3_@rGO-affixed conventional screen-printed carbon electrode (SPCE) was found to exhibit tremendous electrocatalytic capability toward o^8^dGuo oxidation. The amperometric detection of o^8^dGuo worked in the linear range of 50 nM to 315.3 μM, with a LOD of 1.02 nM. The initial sensor response current was still retained after 3000 s. The storage stability was monitored every day for 10 successive days, and no significant deterioration was observed. The method worked well in real human urine and blood serum samples and the results were validated by the HPLC method as well. 

##### GCE Coated with Multi-Walled Carbon Nanotubes (MWCNTs)

**Figure 2:**
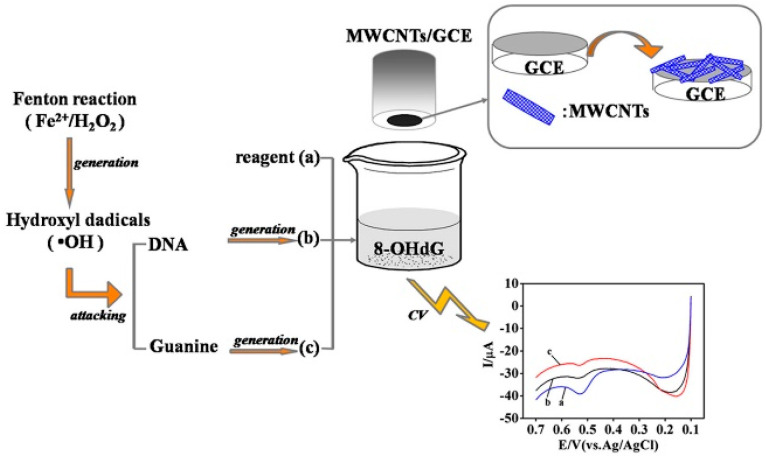
Glassy carbon electrode (GCE)/multi-walled carbon nanotubes (MWCNT) sensor for the determination of o^8^dGuo. Reprinted with permission from Reference [28]. © 2016, Elsevier.

Guo et al. [28] have developed an OG sensor based on the multi-walled carbon nanotubes (MWCNTs)-modified GCE characterized by SEM and EIS methods (Figure 2). The linear ranges were 56.3 nM to 6.08 µM and 6.08 µM to 16.4 µM respectively, with the LOD of 18.8 nM (S/N = 3) in CV measurements. UA and dopamine (DA) commonly coexist with o^8^dGuo in human metabolism and their electrochemical oxidation potentials are close, but with this method, their mixture gave three clear and well-separated oxidation peaks at 0.43 V (o^8^dGuo), 0.36 V (UA) and 0.22 V (DA), respectively.

##### GCE Coated with Porous Single-Walled Carbon Nanotube (PSWCNT)

**Figure 3:**
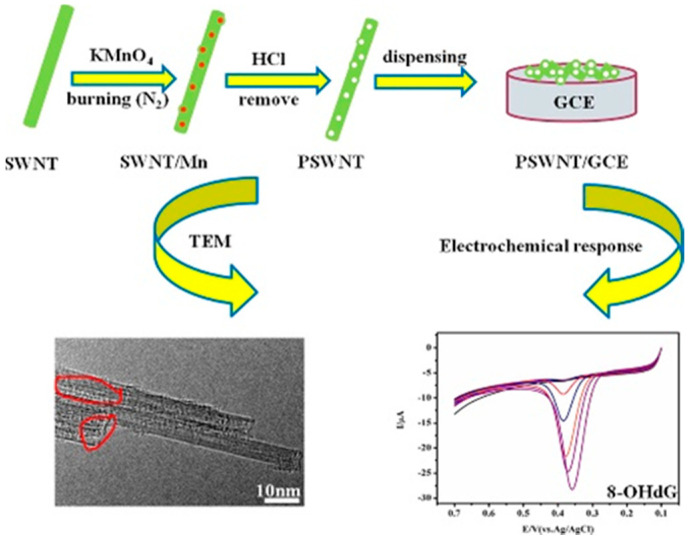
The application of porous single-walled carbon nanotube (PSWCNT) for the determination of o^8^dGuo. Reprinted with permission from Reference [29]. © 2018, Elsevier.

Quantitative measurement of o^8^dGuo concentration was analyzed by linear sweep voltammetry (LSV) as well, by Shang et al. [29]. First, a porous carbon nanotube was obtained from SWCNT using KMnO_4_ as the etching agent, dropping the aqueous PSWCNT onto a polished GCE surface. The obtained PSWCNT/GCE-based sensor, characterized by TEM, X-ray diffraction and nitrogen adsorption/desorption isotherm, showed outstanding electrochemical performance for o^8^dGuo in the measurement ranges 2.99 nM to 3.061 μM and 3.061 μM to 87.25 μM respectively, with an LOD at 1.0 nM. The effects of temperature and time on DNA damage have also been investigated and important dynamical parameters and a kinetic equation with reaction rate constant (k = 2.090 min^−1^) and the apparent activation energy (E_a_ = 30.64 kJ·mol^−1^) for the OG oxidation have been obtained (Figure 3).

##### Voltammetric Sensor for Oxidized DNA Using Ultrathin Films of Osmium and Ruthenium Metallopolymers

In an earlier study, Mugweru et al. [30] assembled films containing the metallopolymers (Os(bpy)_2_(PVP)_10_Cl)^+^ and (Ru(bpy)_2_(PVP)_10_Cl)^+^ (bpy = 2,2’-bipyridine; PVP = poly(vinylpyridines)), layer by layer, on pyrolytic graphite electrodes to obtain sensors that selectively detect oxidized DNA. Assembly of films was monitored with a quartz crystal microbalance (QCM). The two metallopolymers behaved electrochemically independent of each other in the films. This combination provided a catalytic Os square wave voltammetry (SWV) peak that is mainly selective for OG and the detection of other oxidized nucleobases from the Ru peak. The method is applicable to measurements on DNA in solution or DNA incorporated into films. Using the Os SWV peak, a single oxidized nucleobase out of 6000 nucleotides was detected. A related Os-PVP polymer with higher oxidation potential can generate electrochemiluminescence (ECL) with oligonucleotides containing OG in thin films, providing an alternative method to detect oxidative stress [31]. This purely voltammetric approach is complementary to the ECL method.

##### Copper-Based Metal Organic Framework Nanoparticles Anchored to Graphite Nanosheets

**Figure 4:**
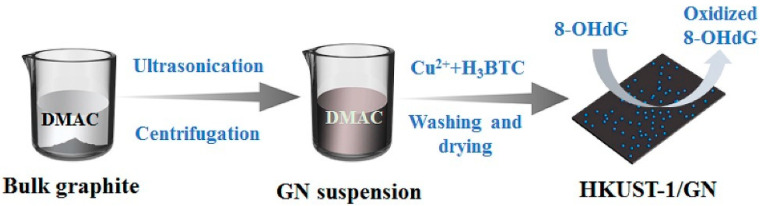
The schematic representation of metal organic framework nanoparticles for the measurement of o^8^dGuo. Reprinted with permission from Reference [32]. © 2019, Elsevier.

Cao et al. [32] prepared graphite nanosheets (GN) in a very simple liquid-phase exfoliation of graphite in *N,N*-dimethylacetamide (DMAc). Ultra-small (less than 5 nm) Cu-based metal organic framework (HKUST-1) nanoparticles were in situ anchored on the surface of GNs with a high degree of dispersion (Figure 4). The synthesized hybrids of graphite nanosheets (HKUST-1/GN) decorated with HKUST-1 nanoparticles, characterized by powder X-ray diffractometry (XRD), thermogravimetric analysis (TGA), SEM and TEM, showed excellent electrochemical sensing performance towards the DNA damage biomarker o^8^dGuo. The DPV concentration measurement was characterized with a fast detection speed (~240 s), wide linear window (10 nM–1 µM), high sensitivity (346,857 µA mM^−1^ cm^−2^), low LOD (~2.5 nM) and good reproducibility.

#### 3.2.2. Gold Electrodes with o^8^dGuo-Specific Aptamers

**Figure 5:**
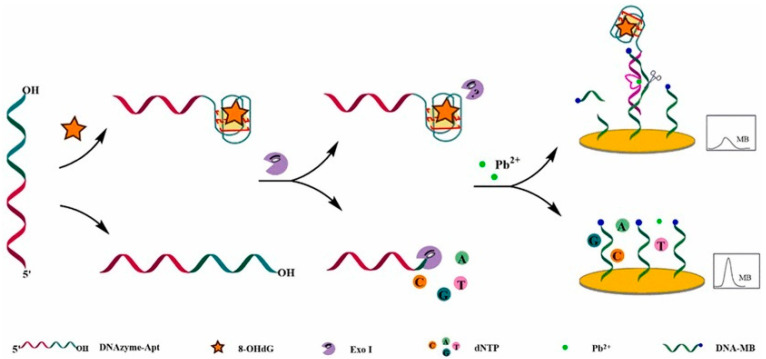
A o^8^dGuo-specific aptamer construct. Reprinted with permission from Reference [33]. © 20019, Elsevier.

An electrochemical method, developed by Zheng et al. [33], combines o^8^dGuo-specific aptamer (apt) with metal ion-dependent DNAzymes and exonuclease to achieve high sensitivity with a LOD of 6.82 pM and linearity from 0.01 nM to 7.0 μM applying SWV. The electrochemical sensing platform used a gold electrode modified with substrate DNA of DNAzyme, labelled with methylene blue (MB) redox probe as a working electrode. The state of the surface of the gold electrode was checked employing EIS. During the SWV analysis, reduction of the electrochemical signal is measured thanks to the release of the MB during the cleavage of the substrate DNA by the DNAzyme part of the o^8^dGuo–apt–DNAzyme complex. In addition, CV was used to verify the experimental principle. The modified gold electrode is stable for a week at 4 °C (Figure 5).

**Figure 6:**
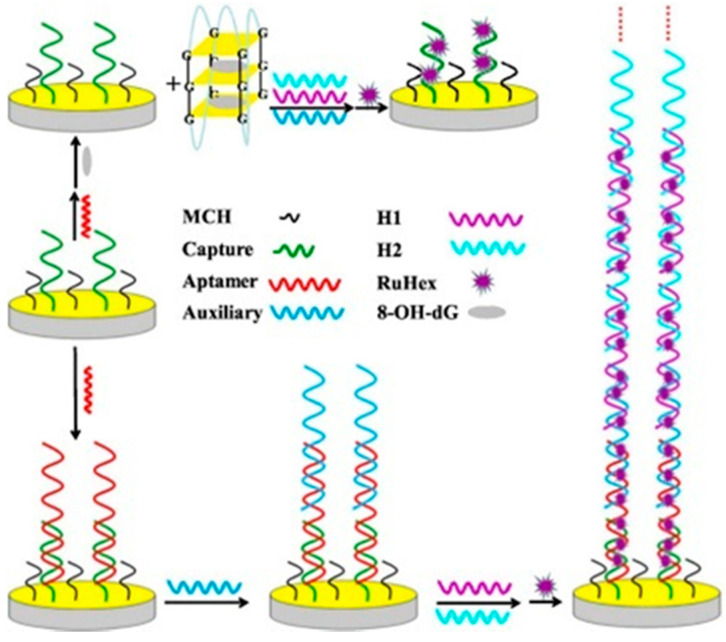
An aptamer-based electrochemical biosensor with hybridization chain reaction (HCR) signal amplification for the detection of o^8^dGuo. Reprinted with permission from Reference [34]. © 2018, Elsevier.

Jia et al. [34] have developed a highly sensitive and selective electrochemical aptasensor by using o^8^dGuo-specific aptamer hybridized with the capture of DNA immobilized on a gold electrode with a sticky tail left, which initiated the hybridization chain reaction (HCR) through an auxiliary DNA (Aux). The formation of extended double-stranded DNA (dsDNA) structure intercalated a more electroactive species ((Ru(NH_3_)_6_)^3+^), therefore the high sensitivity of the electrochemical method was further increased thanks to the HCR. In the presence of o^8^dGuo, the aptamer will form a GQ structure that stops the HCR, leading to detection signal decrease. By monitoring the change in the current of the (Ru(NH_3_)_6_)^3+^ ion, the concentration of o^8^dGuo can be indirectly determined in the sample. The strategy was characterized by EIS measurements. Thus, the resistance continuously increased when the electrode was incubated with capture DNA, 6-mercapto-1-hexanol (MCH), aptamer and Aux. The resistance of aptamer/MCH/capture/electrode heavily decreased in the presence of o^8^dGuo. In DPV, the electrochemical signal was mainly implemented by (Ru(NH_3_)_6_)^3+^ immobilized on the long-nicked DNA polymers. An impressively wide linear response ranging from 10 pM to 100 μM and LOD of 2.5 pM has been attained (S/N = 3). The method was successfully applied in human urine samples (0.56 nM concentration of o^8^dGuo was determined) and interference with UA has been eliminated. The aptasensor was stable for 10 days at 4 °C. Despite the vulnerability of these electrodes, they constitute one of the most specific methods for the quantitation of o^8^dGuo lesions (Figure 6).

#### 3.2.3. Electrochemiluminescence in a Multiple-Mechanism-Driven Biosensor

**Figure 7:**
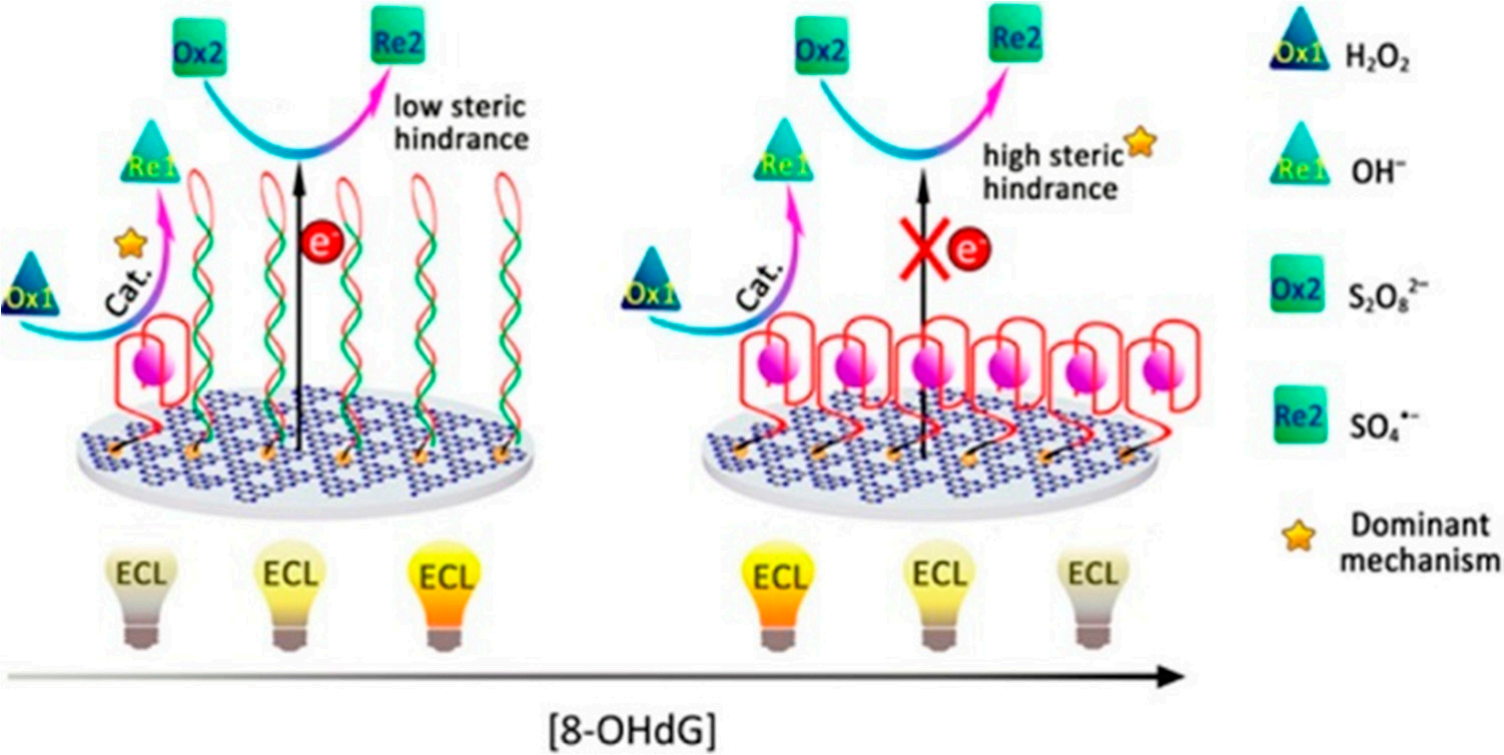
Electrochemiluminescent (ECL) biosensor developed by Lv et al. [35]. Reprinted with permission from Reference [35]. © 2019, American Chemical Society.

Lv et al. [35] have developed a multiple-mechanism-driven electrochemiluminescent (ECL) biosensor that utilizes competitive catalytic and steric hindrance effects by assembling hemin/GQ on carbon nitride nanosheets (CNNS). A hairpin probe, containing a recognition sequence for the aptamer probe and a “caged” GQ sequence, was conjugated to a hybrid of CNNS and gold nanoparticles (AuNPs; CNNS–AuNPs) on GCE. After the targeted o^8^dGuo was captured by aptamer probe (o^8^dGuo-apt), it opened the hairpin structure by hybridizing to it. During the treatment with exonucleases, o^8^dGuo-apts were protected against them by o^8^dGuo and they were released after their complementary sequences were digested. Recycling of o^8^dGuo-apt led to the continuous opening of the hairpin probe and the generation of “active” GQ structures. Finally, by addition of hemin, the liberated GQs were folded into a supramolecular hemin/GQ, that electrocatalytically reduced the H_2_O_2_; as a result, its quenching effect was decreased on the ECL of CNNS–AuNPs. A linear dependence of the sensor on the o^8^dGuo concentration over the range from 10^−15^ to 10^−12^ M with a correlation coefficient of 0.998 was obtained. The LOD was calculated to be 38.8 aM (!), which is much lower than those for the best previously reported biosensors. In human serum spiked with o^8^dGuo, the proposed competitive dual-mechanism-driven biosensor had good accuracy and high precision. The test of the binding specificity of the method indicated that the aptameric recognition function was retained. Many optimizations are required for greater simplicity, lower cost and for instrumentation use (Figure 7).

### 3.3. Optical Methods

#### 3.3.1. Fluorometric Assays

**Figure 8:**
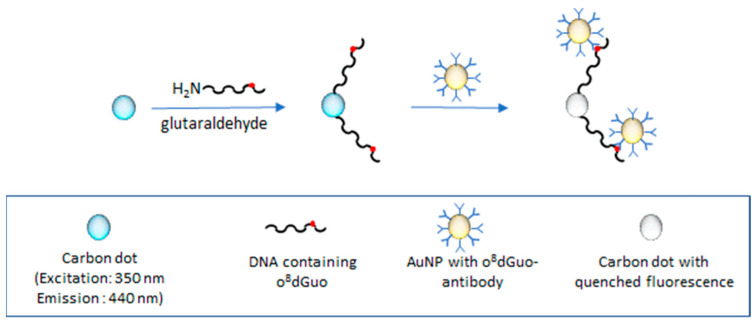
Fluorometric immunoassay using carbon quantum dots (CQDs) for the detection of DNA containing o^8^dGuo lesion [36].

Hu et al. [36] have developed a fluorometric immunoassay, based on strongly photoluminescent and excellently photostable carbon quantum dots (CQDs) and AuNPs, for determining DNA containing oxidatively damaged o^8^dGuo. The morphology and sizes of AuNPs and CQDs were observed by TEM. In a test experiment, CQDs were modified with glutaraldehyde for conjugation of DNA-o^8^dGuo, used as the damaged DNA model. AuNPs were functionalized by o^8^dGuo antibody. The specific reaction of o^8^dGuo and o^8^dGuo antibody brought CQDs and AuNPs into close distance. When the CQDs were excited by UV light, emission of CQDs was quenched by AuNPs. The target DNA-o^8^dGuo was detected by recording the quenched fluorescence spectra with an LOD of 7 pM in the 10 pM to 25 μM DNA-o^8^dGuo concentration range (the paper erroneously gives 700 pM as LOD). The drawback of the method is that it requires DNA-o^8^dGuo isolated from urine and anchored to the glutaraldehyde-modified CQD, while urine contains more free OG than DNA-attached o^8^dGuo (Figure 8).

**Figure 9:**
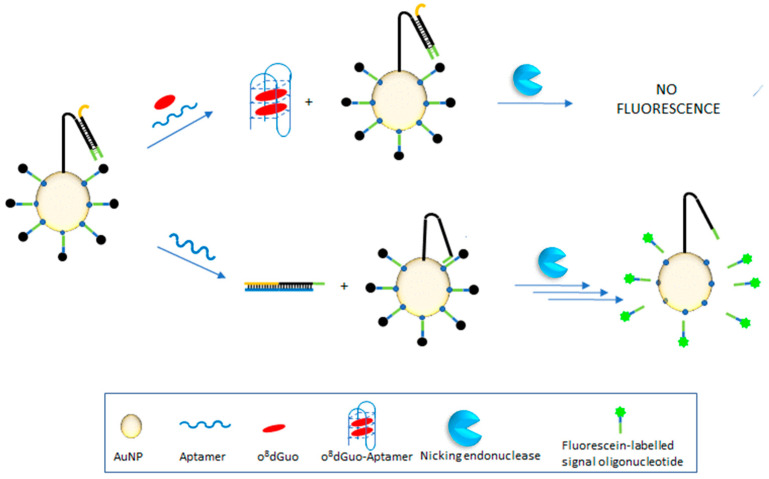
Three-dimensional (3D) nanomachine-assisted detection of o^8^dGuo lesions [37].

Wei et al. [37] developed an ultrasensitive method based on fluorometric determination of o^8^dGuo by using a three-dimensional (3D) DNA nanomachine. The nanomachine was constructed by assembling hundreds of carboxyfluorescein-labeled single-stranded DNA (ssDNA) oligonucleotides (acting as signal reporter, the latter were quenched by AuNPs until the protecting DNA were present) and tens of swing arms (acting as single-foot DNA walkers) on AuNPs. The activity of this DNA nanomachine was controlled by introducing the protecting oligonucleotides. In the presence of an aptamer against o^8^dGuo, the protecting oligonucleotides are removed from the swing arms due to DNA strand displacement reaction. In the next step, the detached DNAwalker (DW) hybridizes to the labelled DNA so that the DNA nanomachine becomes activated. Special sequences of signal reporter in the formed duplex can be recognized and cleaved by nicking endonuclease (NEase). This process gives an energy input for the DW to autonomously and progressively move along the surface of the AuNP, to release hundreds of signal reporters causing a rapid increase in green fluorescence. This 3D nanomachine detects o^8^dGuo in concentrations as low as 4 pM because one aptamer can release hundreds of signal reporters. The linear response range extends from 0.02 to 70 nM. The method worked in the diluted human serum (1:10 ratio) samples spiked with various concentrations of o^8^dGuo. This method is not as fast as the previously reported method based on aptamer and unmodified AuNPs [38] because of several incubation procedures, but the LOD of this method is lower by nearly three orders of magnitude than that of earlier methods (Figure 9).

#### 3.3.2. Visual Analysis of Samples without the Use of Expensive Instruments

**Figure 10:**
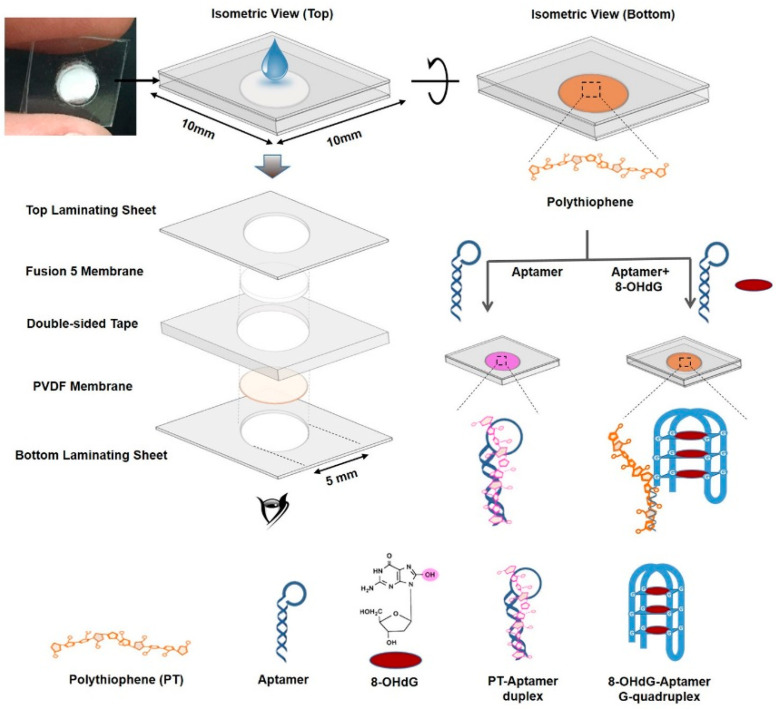
A luminescent paper-based device for the visual detection of o^8^dGuo. Reprinted with permission from Reference [39]. © 2018, American Chemical Society.

Ammanath et al. [39] have reported a naked eye detection of o^8^dGuo by a luminescent paper-based device using a membrane impregnated with an aptamer, as an o^8^dGuo recognition element, and poly(3-alkoxy-4-methylthiophene (PT)), a dye that changes its optical properties upon interaction with an aptamer in the presence/absence of o^8^dGuo (Figure 10). 

The mechanism is that o^8^dGuo induces a conformational change of the aptamer containing a guanine-rich nucleic acid sequence to form GQ structures. This rigid structure reduces the electrostatic interactions between the PT and the aptamer, thereby leading to fluorescence and color recovery of PT. Fluorometric and colorimetric monitoring revealed linear responses for o^8^dGuo concentrations between 500 pM and 500 nM, with LOD of ∼300 pM (fluorescence) and ∼350 pM (colorimetric), respectively (S/N = 3). Colorimetric responses in artificial urine samples allowed rapid, sensitive and selective detection of o^8^dGuo at clinically relevant (pM to nM) concentration levels. The main advantage of this method is that point-of-care early diagnosis of oxidative stress can be done without instrumentation.

**Figure 11:**
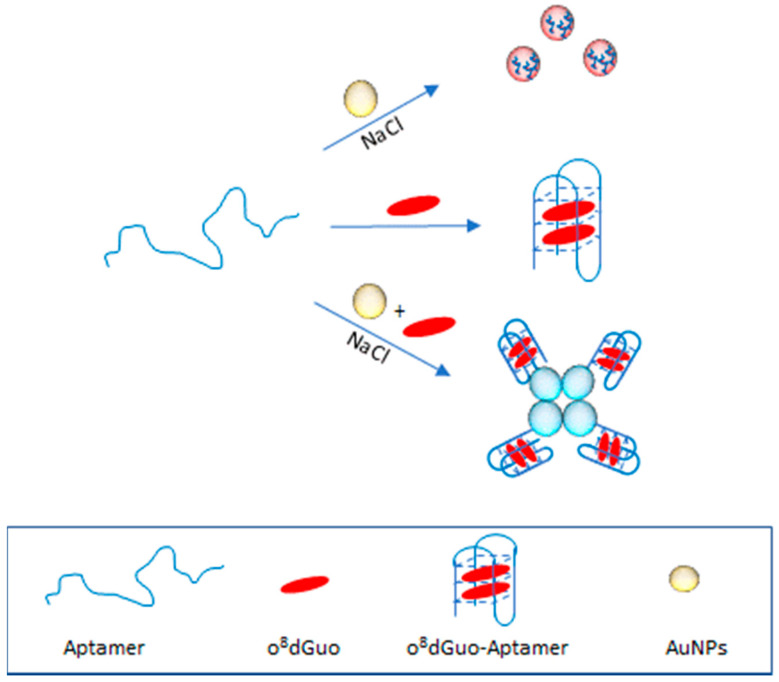
Gold nanoparticles used in the colorimetric determination of o^8^dGuo [38].

A similarly sensitive and specific colorimetric method has been elaborated earlier by Wang et al. [38] for urine sample analysis. The method consists of three steps: (1) the aptamer was adsorbed on the surface of AuNPs which enhances their stability, (2) upon addition of o^8^dGuo, the conformation of the aptamer changes to form a GQ structure and (3) as a consequence, it loses the ability to protect the nanoparticles and causes a color change from red to blue. The conformational changes were also studied by circular dichroism (CD). The response is linear in the range from 5.6 to 282 nM, LOD is 1.7 nM (Figure 11).

#### 3.3.3. Resonance Light Scattering Aptasensor Based on Magnetic Nanoparticles

**Figure 12:**
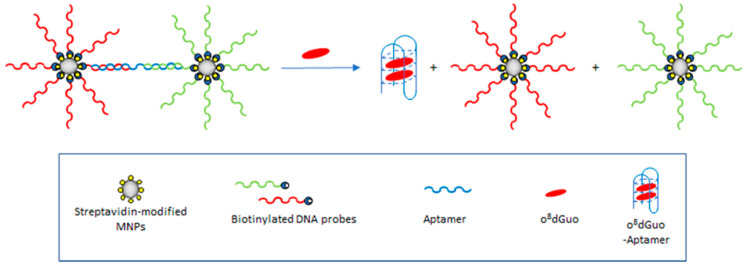
Magnetic nanoparticles in the resonance light scattering (RLS) probe for the detection of o^8^dGuo [40].

Magnetic nanoparticles (MNPs) were employed as resonance light scattering (RLS) probes [40]. The probe DNA was placed on the surface of MNPs, which produces a rather low RLS signal. If, however, the probe DNA hybridizes with the aptamer against o^8^dGuo, a sandwich structure will be formed. This results in a significant enhancement of RLS intensity. The aptamer was used as the recognition element to capture o^8^dGuo. o^8^dGuo has a stronger affinity for the aptamer than probe DNA, and the conformation of the aptamer therefore switches from a double-stranded to a GQ structure. As a result, MNPs labeled with probe DNA are released, and RLS intensity decreases. The method allows o^8^dGuo to be detected with a linear response in the 32 pM−12.0 nM concentration range and with a LOD of 11 pM. The MNPs can be reused five times by applying an external magnetic field for recycling. The method was successfully applied to analyze human urine samples for its content of o^8^dGuo. It was also found that the levels of o^8^dGuo noticeably increased with the increase of the Air Quality Index, and the method is a viable tool to investigate the relationship between o^8^dGuo levels and the effect of air pollution. o^8^dGuo removal is tightly regulated by external and internal stimuli, e.g., ROS accumulation that is affected by air pollution as well (Figure 12) [7].

#### 3.3.4. UV Radiation

**Scheme 2:**
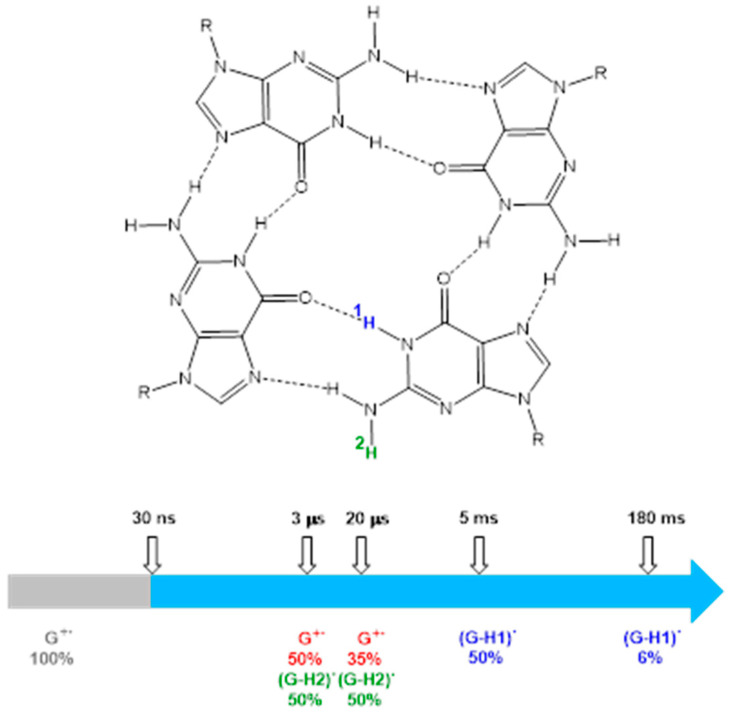
Effect of UV irradiation on G transformations. Adapted with permission from Reference [41]. © 2017, American Chemical Society.

Banyasz et al. [41] have carried out the first study on oxidative damage of human telomere GQs without the mediation of external molecules. The investigation has been performed for GQs formed by folding of heneicosamer d(GGG(TTAGGG)_3_) single strands in buffered solutions containing Na^+^ cations (TEL21/Na^+^). Coupling nanosecond time-resolved spectroscopy and quantum mechanical calculations (TD-DFT), the study focused on the primary species, ejected electrons and guanine radicals, generated upon absorption of UV radiation directly by TEL21/Na^+^. At 266 nm, corresponding to an energy significantly lower than the guanine ionization potential, the one-photon ionization quantum yield was 4.5 × 10^−3^, this value is comparable to that of cyclobutane thymine dimers, the major UV-induced lesions in genomic DNA (quantum yield = (1.1 ± 0.1) × 10^−3^). The fate of guanine radicals, generated in an equivalent concentration with that of ejected electrons, has been followed over five orders of magnitude of time. Such a quantitative approach reveals that an important part of radical cation population survives up to a few milliseconds, whereas radical cations produced by chemical oxidants in various DNA systems are known to deprotonate, at most, within a few microseconds. Under the same experimental conditions, neither one-photon ionization nor long-lived radical cations are detected for the telomere repeat d(TTAGGG) in single-stranded configuration, showing that secondary structure plays a key role in these processes. Two types of deprotonated radicals are identified: on the one hand, (G-H2)^•^ radicals (guanine radical deprotonated at the amino group), stable at early times, and on the other hand, (G-H1)^•^ radicals (guanine radical deprotonated at N1, crucially involved in GQ formation), appearing within a few milliseconds and decaying with a time constant of ∼50 ms (Scheme 2). HPLC-coupled mass spectrometry also revealed the presence of o^8^dGuo; however, o^8^dGuo is a minor oxidation product in telomeric GQs, the quantum yield of o^8^dGuo formation ((3.2 ± 0.3) × 10^−4^) is only 7% of the one-photon ionization quantum yield. Still, the quantum yield for o^8^dGuo formation in telomeric GQs is one order of magnitude higher than that determined for naked genomic DNA [42].

### 3.4. Hyphenated Techniques

#### 3.4.1. Gas Chromatography–Mass Spectrometry (GC–MS) Analyses

Rozalski et al. [43] have investigated the formation of urinary 5-hydroxymethyluracil (hm^5^Ura) and OG as potential biomarkers in patients with colorectal cancer using the GC–MS method. These two biomarkers have greater predictive value as a cancer biomarker when used in combination (urinary OG and o^8^dGuo together with hm^5^Ura) but are still insufficient as an ideal marker. It is likely that these biomarkers filtered out subjects of the study with higher oxidative stress/chronic inflammation and with a lower ability to remove the deleterious hm^5^Ura:G mispair. It is also noteworthy that the measurement of DNA lesions in urine can be a good non-invasive biomarker for oxidative stress assessment in colorectal cancer. However, diagnostic performance of this method for early detection is moderate.

#### 3.4.2. G Oxidation Study by EC-LC-MS Chromatography Technique

The EC-LC-MS method integrates electrochemical (EC) oxidation with liquid chromatographic (LC) separation and mass spectrometric (MS) detection. Oberacher et al. [44] have studied the oxidation behavior of nucleic acid derivatives using this approach. It was found that DNA nucleosides were more stable than RNA nucleosides, the electrochemical stability increased in the order guanine ≈ acyclovir ≈ Guo < dGuo ≈ d(GG) < d(GGG). The mechanistic studies have involved the oxidation of the dinucleotide d(GG) in more detail. In particular, different forms of inter-strand cross-links (d(GG)-H)_2_, intra-strand cross-links (d(GG)-2H) as well as mono- and di-hydroxylated species ((d(GG) + O), d((G + O)(G + O)) were detected. d(GG) is first oxidized at a G in a one-electron process to the corresponding radical. Two radicals are combined to form cross-links of the form (d(GG)-H). Intra-strand cross-links were produced at more positive electrochemical potentials than the inter-strand cross-links. In MS/MS, only fragments originating from the consecutive cleavages of the linked bases from the backbone were observed. The main fragmentation reactions of (d(GG)-H_2_ + 2H)^2+^ were cleavage of the C1′-N9 bond between the sugar and the nucleobase, cleavage of the sugar-phosphate backbone at C3′-O and combinations thereof. Especially, the simultaneous observation of (G + H)^+^ and ((G-H)_2_ + H)^+^ was a clear hint for linking two Gs from two different molecules. Hydroxylation of the G residues gave rise to the formation of d(GG) + O, which is further oxidized to d((G + O)(G + O)) species. The averaged fragment ion mass spectrum of the d(GG) + O forms shows that fragment ions were produced by consecutive cleavages of C1′-N9 and C3′-O bonds, respectively. The formation of directly cross-linked Gs have not been observed in genomic DNA as products of oxidative damage. Only the formation of G-cytosine (G(8–5)C) as well as G-thymine (G(8–3)T) intra-strand cross-link lesions was confirmed (the numbers in brackets denote the interlinked atoms between the pertinent nucleobases). The EC-LC-MS method is well suited for the study of oxidation reactions of short oligonucleotides.

#### 3.4.3. Aptamer-Based Online Magnetic Solid Phase Extraction for HPLC–MS Analysis

**Figure 13:**
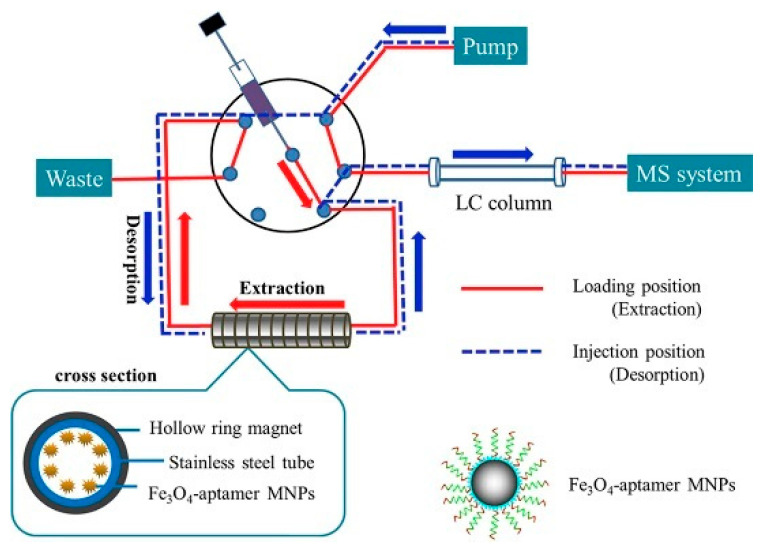
An aptamer-based online magnetic solid phase extraction for HPLC–MS analysis of o^8^dGuo. Reprinted with permission from Reference [45]. © 2018, Elsevier.

Gan and Xu [45] have developed a novel aptamer-based online magnetic solid phase extraction (MSPE) method for the selective determination of o^8^dGuo in human urine (Figure 13). Magnetic aptamer adsorbent (Fe_3_O_4_-aptamer MNPs) was synthesized for the selective extraction of o^8^dGuo by crosslinking amino-functionalized-Fe_3_O_4_ with an o^8^dGuo aptamer by glutaraldehyde and fixed into a stainless-steel tube as the sorbent of MSPE. After selective extraction by the aptamer adsorbent, the adsorbed o^8^dGuo was desorbed dynamically and analyzed online by HPLC–MS. The synthesized sorbent presented outstanding features, including specific selectivity, high enrichment capacity, stability and biocompatibility. Moreover, the proposed MSPE–HPLC–MS method can achieve adsorption and desorption operation integration, greatly simplify the analysis process and reduce human errors. When compared with offline MSPE, a sensitivity enhancement of 800 times was obtained for the online method. Some experimental parameters, such as the amount of the sorbent, sample flow rate and sample volume, were optimized systematically. Under the optimal conditions, low limit of detection (35.3 pM, S/N = 3), limit of quantity (106 pM, S/N = 10) and wide linear range (177 pM–706 nM) were obtained for the method and with good recoveries of o^8^dGuo in urine samples (82–116%). The method is simple, and the extraction, desorption and separation steps can be completed within 11 min. This selective, sensitive and automated method is expected to become a potential approach for the selective determination of trace o^8^dGuo in complex urinary samples (Figure 13).

### 3.5. Summary and Comparison of the Efficacy of Analytical Methods

**Figure 14:**
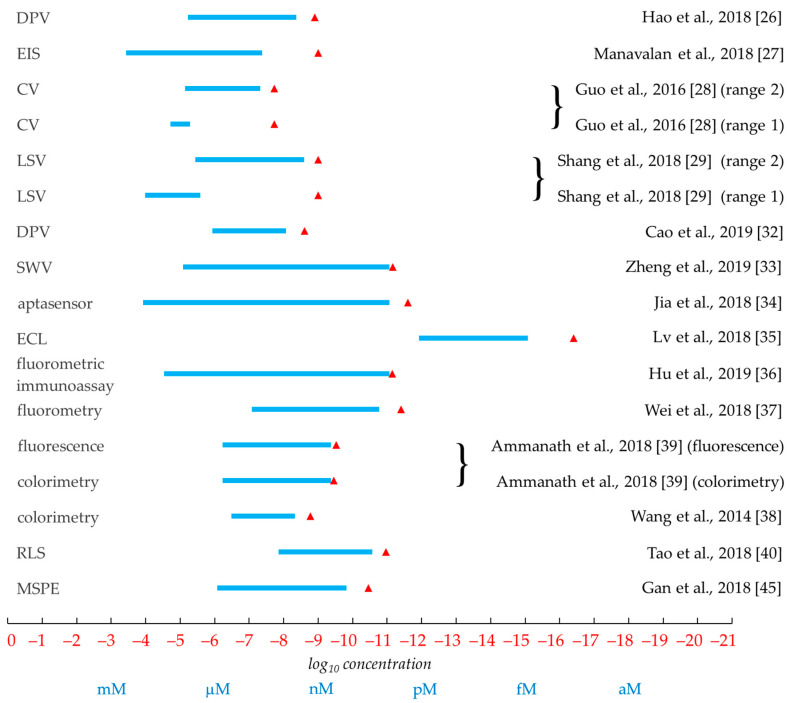
Measurement ranges (**blue bars**) and LOD values (**red triangles**) of quantitative analytical methods. The quantitation methods are listed on the left, the references on the right side.

The performance of the individual analytical methods for the characterization of oxidative lesions shows great variation in terms of methods used, compounds detected, nature of analytical constructs, robustness, timeliness, accuracy, types of information obtained, detection range and sensitivity (Table 1). Several methods operate with samples in bodily matrices (e.g., urine, blood, serum) where interference with other substances (e.g., UA, DA) must be eliminated. Regarding the sensitivity, on the low end, some methods embrace 2–3 orders of magnitude, while on the high end, we may encounter methods that are able to span 6–7 orders of magnitude (Figure 14). In terms of sensitivity, even the least sensitive methods are in the low nanomolar range, while the record holder method can boast of a near-attomolar value (Figure 14). In general, both the electrochemical and the optical methods offer a good choice of options for the determination of oxidative lesion detecting either the nucleoside o^8^dGuo or the damaged ON. It is noteworthy that beyond direct detection, sample enrichment also can contribute to signal enhancement [45]. Beyond the information on the quantity, concentration or frequency of oxidative lesions, in some cases, additional pieces of information can hint at temperature-dependence, kinetic and thermodynamic parameters [29], processes preceding oxidation [41] or eventual cross-link formation in short ON tracts [44]. The transformation of G can take place in different sequence contexts, including GQs, which have also been utilized in some analytical methods [34,35,38,39,40].

## 4. Structural Studies and Assays for Investigation of Structure–Function Relationship

These studies aim not only at the quantitation of OG sites in a specific nucleic acid region, but also at the determination of the position of OG modification sites in the sequence and analyzing the effects of these modifications on the secondary and 3D structure of the examined DNA region. Furthermore, these techniques together with in vitro and in vivo functional assays are designed to investigate how the oxidation of Gs in the promoter regions can regulate the expression level of certain genes involved in the repair mechanism via the structural changes of these GQ motifs.

### 4.1. Nanopore Detection

**Figure 15:**
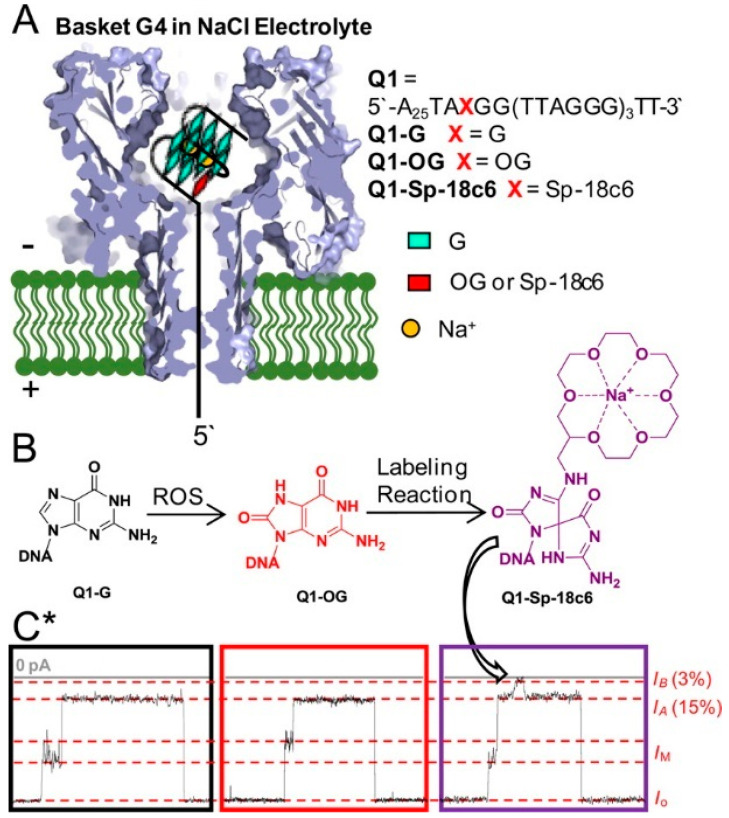
The schematic representation of α-hemolysin (α-HL) nanopore technology to detect OG. (**A**) Scheme for experimental setup, including the G4 sequence studied and location of the OG and Sp-18c6-labeled OG. (**B**) Reaction scheme for G conversion to OG by ROS, followed by labeling of OG with aminomethyl-18c6 (18c6) in the presence of the mild one-electron oxidant K2IrBr6 to furnish the labeled OG product, Sp-18c6. (**C**) Current vs time traces for Q1-G (black box, left), Q1-OG (red box, middle), and Q1-Sp-18c6 (purple box, right). Reprinted with permission from Reference [46]. © 2015, American Chemical Society.

The Burrows group [46] has applied the α-hemolysin (α-HL) nanopore technology to detect and quantify OG in the human telomeric DNA sequence as protein nanopores can detect DNA solutes through multiple recognition zones that are specific for sequence and secondary structure. The detection of OG with α-HL required labeling of OG with an aminomethyl-functionalized 18-crown-6 using a mild oxidant to generate the spiroiminodihydantoin derivative (Sp) suitable for the coupling (Figure 15). The labeled OG yielded a pulse-like signal in the current versus time trace when the DNA strand was electrophoretically passed through α-HL pore in NaCl electrolyte. To increase the rate of translocation a mixture of NH_4_Cl (100 mM) and LiCl (2 M), electrolytes induced the GQ propeller fold to unravel quickly outside the α-HL channel. This electrolyte mixture allowed the observation of the labeled OG, while providing a faster recording of the currents. In addition, OG distributions were probed with this method in a 120-mer stretch of the human telomere sequence (5′-d(TTAGGG)-3′ repeats) exposed to the cellular oxidant ^1^O_2_. Single-molecule profiles determined the OG distributions to be random in this context. The α-HL nanopore approach could be feasible for quantification of OG and determination of telomere length in a single experiment. Subsequently, the same group [47] has extended this method to differentiate G:C versus A:T and G:C versus G:m^5^C base pairs (m^5^C = 5-methylcytosine) in the latch zone of the α-HL channel, a previously unrecognized sensing zone specific for dsDNA structure [48]. It was found that the most sensitive region of the latch can readily discriminate duplexes in which one G:C base pair is replaced by an A:T pair. Additional experiments determined that while neither OG nor 7-deazaguanine (c^7^G) opposite C could be differentiated from a G:C base pair, in contrast, the epigenetic marker m^5^C, when present in both strands of the duplex, yielded new blocking currents when compared to strands with unmodified cytosine. This result was based on earlier studies that demonstrated the possible distinction of the abasic site versus G:C base pair positioned in the latch zone at the top of the α-HL vestibule [48,49,50].

Nanopore sequencing of oligonucleotides is a promising method, yet its robustness still needs improvement [51]. Endogenous level of OG in genomic DNA is relatively low and may not be easily detected by Sanger sequencing. Tang et al. [52] have observed that H_2_O_2_ treatment induces the formation of OG at the 5′-dG in the d(GG) motif and at the middle dG of the d(GGG) motif. They were able to locate these OG lesions in the genome using two commercially available DNA polymerases, *Bsu* DNA polymerase (*Bsu* Pol) and *Tth* DNA polymerase (*Tth* Pol), which can selectively incorporate adenine (A) and cytosine (C) opposite OG, respectively. Parallel analysis of the primer extension products with *Bsu* Pol and *Tth* Pol followed by sequencing provided quantitative detection of OG at single-base resolution in synthesized DNA as well as in the G-rich telomeric DNA from HeLa cells. 

### 4.2. NMR and CD Spectroscopy and T_m_ Studies to Investigate the Secondary and 3D Structure of GQs

#### 4.2.1. NMR Spectroscopy

1D ^1^H-NMR spectroscopy is used for the simplest determination of GQ formation. The usual conditions involve the use of potassium phosphate buffer at pH 7–7.4 containing 50–100 mM KCl salt in 10% D_2_O running a WATERGATE solvent suppression pulse sequence. It allows the observation of the G:G Hoogsteen imino peaks in GQs at 11–12 ppm [3,53]. The analyses of GQ structures in the NEIL3 promoter sequence [3] showed that the OG modifications can restrict the probable number of GQ conformations, resulting in sharper imino peaks in the spectra compared to the natural sequence having less resolved peaks due to the mixture of different GQ folds. On the other hand, multidimensional NMR experiments are used for the deeper elucidation of quadruplex structures [54,55].

#### 4.2.2. CD Spectroscopy

A typical condition of CD spectroscopy experiments for the analyses of GQ structures include 20 mM cacodylic acid (pH 7.4), 140 mM KCl and 12 mM NaCl. For K^+^-dependent experiments, potassium phosphate buffer is used (pH 7) with the concentration of K^+^ ranging from 1 to 200 mM. Along with the NMR experiments, the shape of CD curves (negative and positive bands at 240/260 nm respectively, and broad shoulders at 290 nm) can indicate the type of GQ folds (parallel, antiparallel, hybrid) in the examined cases [3]. The technique can also be used for the investigation of the *syn*/*anti*-conformation of an individual guanine in the quadruplex by the replacement of the questioned guanine with 8-bromoguanine and comparing the CD spectrum of the native sequence with the modified one. In case of the CD curve changing with the modification, the targeted guanine position had an anti-conformation as the 8-bromoguanine forms *syn*-conformation [53].

#### 4.2.3. Melting Temperature (T_m_) Analysis and Its Combination with 8-Bromoguanine Scan

The simplest analysis of thermal stability of GQs is the measurement of the T_m_ value. Similar to the CD spectroscopic experiments, it is also measured either in a 20 mM cacodylic acid buffer (pH 7.4) containing the 140 mM KCl and 12 mM NaCl or potassium phosphate buffers are used (1–200 mM) for potassium-dependent experiments. A 5 µM oligonucleotide sample containing solution is heated to 100 °C, cooled to 20 °C (at 1 °C/min rate) and the absorbance is monitored at 295 nm in both the increasing and decreasing direction of the temperature. Although the most important information of T_m_ value is the fact that the interrogated DNA sequence can or cannot form a stable quadruplex structure at 37 °C (e.g., having 70 °C T_m_ value), the T_m_ measurement coupled with the 8-bromoguanine scan [56,57,58,59] can give information about the *syn*/*anti*-conformations in a GQ structure as well. If a specific guanine is replaced with 8-bromoguanine, which is known to strongly favor the *syn*-conformation, the destabilizing effect of the change can suggest the *anti*-conformation of the original guanine. The decrease in the T_m_ value is usually only 2–3 °C but the change in the CD spectrum can also support the confirmation of the anti-conformation or the *syn*-conformation when no significant change is observed [3,53,60].

### 4.3. Functional in vitro and in vivo Assays to Understand the Role of Oxidative Damage of GQs in Gene Regulation

#### 4.3.1. The Response of GQs to Oxidative Damage

Omaga et al. [3] have found that the fifth domain in the GQ-forming sequence of the human NEIL3 promoter locks DNA folding in response to oxidative damage to give OG. Beyond spectroscopic techniques (NMR, CD, thioflavin T fluorescence enhancement and ultraviolet−visible) and T_m_ studies, a DNA polymerase stop assay has been developed to reveal this phenomenon. This polymerase stop assay can be used to determine the intra-strand GQ formation on a given template sequence having 60–70 base length. Usually, a short, radioactive 5′-phosphate-labeled primer is used in a linear amplification step-applying a KCl-containing buffer. As the quadruplex formation is K^+^-dependent, LiCl is applied instead of KCl for the negative control experiments. After the extension reaction is stopped, the products are separated on a denaturing 20% polyacrylamide gel by electrophoresis and the bands are visualized using phosphorus autoradiography. In case of stable quadruplex formation, a truncated DNA strand is produced instead of the full-length product. The method can be used as a functional assay determining potential quadruplex-forming sequences in promoter regions of genes which can act as barriers in transcription, but also applicable to analyze the effect of guanine oxidation in such regions when chemically synthesized, site-specific, OG-containing templates are used. The formation of OG modulates the properties of the NEIL3 G4 when the fifth G track is present to favor a parallel-stranded G4 when OG is in a core position, and an antiparallel fold when OG is in a loop position. These studies further determine that oxidation of G in a critical G-track for GQ formation can be rescued by recruitment of the fifth G track (“spare tire” domain) to lock the GQ structure in a more stable fold.

Zhu et al. [53] have also found that the potential GQ-forming sequences in the promoter region of the human RAD17 gene have the ability to fold to a GQ consistent with a hybrid-like topology under model physiological conditions (this is different from other potential GQ-forming sequences in promoter regions that usually adopt parallel folds). With two extra nucleotides of the native sequence on either side of the GQ, the structure was found to fold into a hybrid-like G4, similar to the hybrid-1 fold that the human telomere sequence can adopt. With only one nucleotide on either side of the potential GQ-forming sequences, the topology of the structure was observed to be mixed, and without extra nucleotides on the ends, the sequence adopted a parallel fold. This sequence was also interrogated with synthetic incorporation of the oxidative modification OG into specific sites and installed into the promoter of plasmids with a luciferase gene. These plasmids were transfected into a human cell line to observe the effect of the G4s on transcription. The potential GQ-forming sequence RAD17 was found to decrease luciferase expression with the presence of OG that is consistent with RAD17 expression under oxidative stress.

#### 4.3.2. OG–Xanthine Tetrads in Quadruplex Structures

**Scheme 3:**
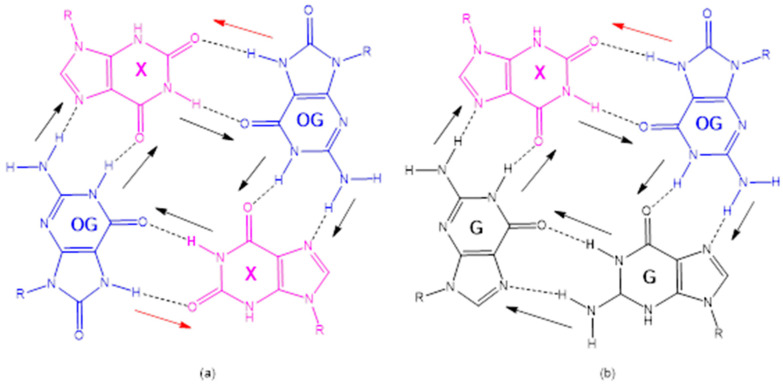
Tetrads containing (**a**) two or (**b**) one X:OG units. The arrows denote the orientation of hydrogen bonds.

The altered base–base recognition as a result of oxidatively modified guanine might be the source of possible mutations. Combination of OG with xanthine (X), another important base lesion derived from guanine deamination, was found to allow the formation of peculiar tetrads containing an X:OG base pair. In their pioneering study, Benz and Hartig [61] have examined the effect of X:OG:X:OG tetrads on GQ stability in a 24nt vertebrate telomeric repeat sequence {d[TAG_3_(T_2_AG_3_)_3_T]}. Four sequences were synthesized that each contained two OG and two X modifications instead of G (Scheme 3a). Gel shift experiments proved the presence of monomolecular quadruplexes, although higher order aggregates were visible as well. Then, CD measurements have been carried out in potassium-containing buffer conditions that are known to favor the presence of an equilibrium of both parallel and anti-parallel topologies of the native telomeric sequence. The CD patterns of modified sequences showed clear shifts to either parallel or anti-parallel topologies, depending on the actual position of modifications. It was also found that incorporation of the above unnatural tetrads slightly destabilizes the quadruplex structure compared to a conventional tetrad made up from guanines (based on CD melting experiments). This phenomenon, in part, can be attributed to the fact that, in comparison to the guanine tetrad, the orientation of the hydrogen bonds is inverted at two positions (cf. Scheme 3a). It is known that adjacent hydrogen bonds with the same direction strengthen (a phenomenon called “cooperative effect”), while those with opposite direction weaken each other [62]. These results suggest that it is possible to program the overall topology of quadruplexes by redesigning constructive hydrogen bonding patterns.

Single point mutation of either OG or X within a G tetrad would result in a loss of a hydrogen bond and/or potential steric hindrance, but double mutation (X:OG or OG:X) in a proper orientation where the hydrogen donor/acceptor pattern points at the Hoogsteen face of OG is complementary with that of the Watson–Crick face of X. Cheong et al. have interrogated the human telomeric sequence {hTel; d(T_2_G_3_(T_2_AG_3_)_3_A)} by replacing nucleotides at specific positions (Scheme 3b) [63]. The simultaneous substitution OG9 (*syn*-conformation) and X17 resulted in a GQ structure that is comparable in thermal stability with the native hTel GQ sequence (ΔT_m_ +1.8 °C). The solution structure of this modification displayed the same (3 + 1) folding topology in both the protonated and deprotonated states (the X residue showed a pK_a_ ~ 6.7). The hTel analogue with a deprotonated X:OG pair was shown to exhibit a more electronegative groove compared to that of the unmodified one, which might have implications in drug binding, DNA–protein interactions and the preparation of nano-scale wires.

The polymorphic topology of GQs is subject to several factors (stoichiometry, strand orientation, types of loop connecting the G-tracts, the glycosidic angle of G-tetrad-forming guanine bases (*syn* or *anti*), the type and concentration of cations, the presence of co-solutes, base modifications, DNA backbone chirality, the sugar puckering, the number of the G-tetrads etc.). Cheong et al. [64] have continued their studies on hTel modifications with an X3:OG9 base pair in the top G-tetrad of this molecule. This is an unfavorable orientation in the original G-tetrad arrangement, however, by inverting the glycosidic orientation of the bases, a G:G:X:OG tetrad with the favorable X:OG pair arrangement could be formed if the hydrogen-bond directionality of the tetrad is reversed. The hTel analogue with top G17:G21:X3:OG9 tetrad adopted an overall fold that is unchanged from the (3 + 1) GQ fold of the original sequence, the polarity inversion of hydrogen-bond directionality resulted in a change in the CD profile. Namely, a negative peak appeared at 240 nm and a positive peak at 265 nm (similar to the CD signature of a parallel G-quadruplex). This modification led to a decreased thermal stability (ΔT_m_ –12 °C) compared to the native hTel ON. The NMR investigation of analogues has been carried out using the characteristic cyclic H1–H8 nuclear Overhauser effect (NOE) patterns by using site-specific ^15^N-labeled samples. The H8–H1′ NOEs proved that the G17:G21:X3:OG9 tetrad adopts a *syn–anti–anti–anti*-conformation while in the native hTel the top tetrad G17:G9:G3:G21 has an *anti–syn–syn–syn*-conformation. The melting temperature of the major form of alternative modification OG17:G21:G3:X9, presumably a fold with OG adopting a *syn*-conformation, is approximately 6 °C higher than that of G17:G21:X3:OG9 modification, in which OG adopts an *anti*-conformation. The CD profile of OG17:G21:G3:X9 analogue is consistent with the formation of multiple GQ conformations with one (presumably the same folding topology as that of G17:G21:X3:OG9 analogue) giving a positive peak at 265 nm and other conformation(s) giving a positive peak at 290 nm.

The X:OG modification in GQ structural studies has also been exploited to shed light on the effect of metal ions and cosolutes on GQ polymorphism. Fujii et al. [65] have studied this problem using the 22nt hTel sequence d(AG_3_(T_2_AG_3_)_3_) with selected modifications in order to stabilize the antiparallel conformation. Thermodynamic analyses of these oligonucleotide analogues have revealed that Na^+^ stabilized the antiparallel GQ, whereas K^+^ destabilized this topology. This result suggests that metal ions selectively stabilize GQ topologies with cavities between G-tetrad planes of certain sizes. In the presence of KCl in 20 wt% poly(ethylene glycol) with average molecular weight of 200, the antiparallel basket-type GQ conformation was not stabilized compared with the dilute condition. In the presence of NaCl, the cosolute did stabilize the GQ with respect to the dilute condition. These data show that strategic substitution with X and OG effectively restricts GQ topology, the ensuing structures depend on sizes of metal ion cavities, hydration states, cosolutes and crowding agents.

Although the X:OG pair would be difficult to generate naturally, this rationally conceived structural element might find applications in the design of GQs with preset structures and constrained topologies that will facilitate their investigation and may be used for therapeutic or nanomaterials applications.

### 4.4. Computational Simulations of Quadruplexes: Effects of OG Incorporation or Apurinic/Apyrimidinic Sites

OG can adopt two alternative conformations (*anti* or *syn*) in the active site of DNA polymerases and therefore OG has dual coding potential in dsDNA. The *anti*-conformation of OG allows Watson–Crick base pairing with a cytosine, whereas the *syn*-conformation of OG forms a stable mispairing with an adenine in a normal *anti*-conformation by Hoogsteen base pair [8,52]. The situation in GQ structures is more complicated, as exemplified by computational investigations that play an emerging role in their examination. In principle, all kinds of calculation techniques can be applied in connection with structural changes based on epigenetic damage, like molecular mechanical, quantum mechanical or mixed quantum and molecular mechanical methods. Interestingly, although numerous theoretical investigations were aimed to study the changes related to DNA damage, like G to OG modification by ROS (see, e.g., Reference [66]), these calculations primarily focused on the core of the effect (e.g., only to the nucleobase part), and therefore, none of them directly considered the full GQ structure. Thus, neither pure quantum-mechanical nor mixed quantum and molecular mechanical investigation was found regarding the full structure in the examined period.

**Scheme 4:**
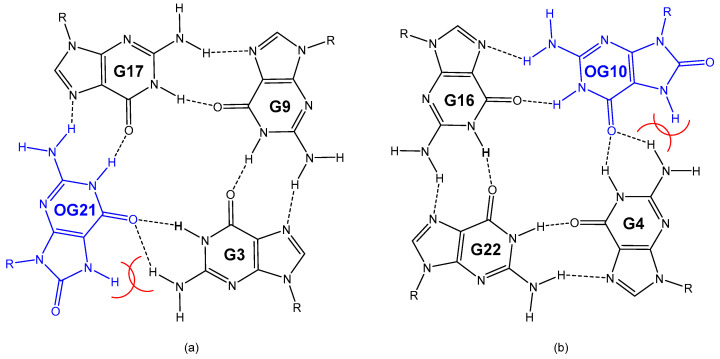
The hydrogen bonding pattern of selected tetrads containing OG units in different layers, (**a**,**b**).

The Plavec group investigated the effect of the presence of OG in different positions within three layers of a GQ structure [67]. They combined NMR measurements with molecular mechanical calculations using simulated annealing conformational sampling techniques. The authors systematically altered single G to OG in ONs, which substitutions provided 12 cases for a three-layered system of 24nt human telomeric (hTel) oligonucleotide sequence model d(T_2_G_3_(T_2_AG_3_)_3_A). According to the NMR measurements, they found stable GQ structures in two cases, where in one of the cases the OG was located in the middle layer (at position 10), while in the other one the OG was in the peripheral layer (at position 21). Considering first the external layer situation, it is important to mention that OG prefers the *syn*-conformation, and since the original substituted G base was also in *syn*-conformation in the native GQ structure, the G to OG change in the external layer could be tolerated by small structural rearrangement in the GQ geometry, the hTel-OG21 structure still adopts a hybrid-1 structure like the original hTel sequence. Molecular dynamical simulations revealed that the steric hindrance between hydrogens in adjacent OG and G units could be eliminated by small outward rotation of the OG base. Consequently, it provided a bifurcated bond at the acceptor side of the OG base contrary to the usual G situation where two hydrogen bonds always formed between adjacent units (G3 > OG21:G17:G9; the sign > denotes a bifurcated hydrogen bond, while the colon sign denotes two single hydrogen bonds, Scheme 4a).

**Figure 16:**
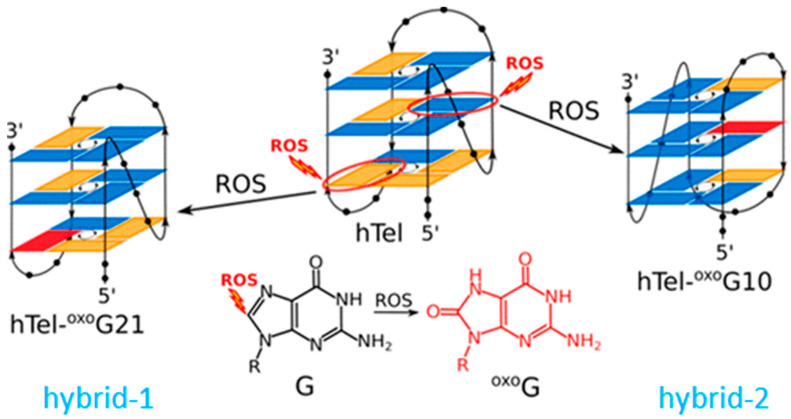
The influence of OG on telomeric topology. Adapted with permission from Reference [67]. © 2019, American Chemical Society.

The simulations also indicated some connection between loop and the external G4 layer; however, these stacking interactions have not been corroborated by NMR measurements. Concerning the middle layer case, the original G was in *anti*-conformation in the three-layered model system, therefore the G10 to OG10 change caused larger backbone rearrangement in GQ structure, the G4 > OG10:G16:G22 (Scheme 4b) layer also presented a bifurcated bond (G4 > OG10) and the hTel-OG10 adopts the hybrid-2 form (Figure 16). Oxidative lesions generally reduce GQ stability, the parent hTel GQ is relatively stable and exhibits a melting temperature of 65 °C, while hTel-OG10 and hTel-OG21 melt at 44 and 49 °C, respectively. This is in line with the earlier observations [1].

Hognon et al. [68] have studied another possible consequence of epigenetic effects, namely the formation of the so-called abasic or apurinic/apyrimidinic (AP) site in the 22nt hTel sequence d(AG_3_(T_2_AG_3_)_3_) GQ structure that was examined by molecular dynamics. They distinguished eight instances according to the position of the AP site in the GQ structure, where four cases were related to the middle layer and four cases to the external layer. When the AP site was in the external layer, it was found that the layer character of the remaining three units was rapidly lost, conceivably a single G base could be found stacking to the middle layer. At the same time, the remaining two G4 layers remained stable without any significant distortion. It is important to note that cations (two K^+^ ions) were always present in the central channel in these simulations. It was found that the K^+^ ions, which were in connection with the AP-containing external layer, were released, while the K^+^ between the two stable layers remained bounded during the whole simulation. The ensuing AP site in the middle layer resulted in a more complex situation. In one case, the whole GQ structure was disrupted, which was naturally accompanied by the release of both K^+^ cations from the central channel. In the other three cases, the GQ structure was kept together in such a way that the cations stayed bounded in the central channel, and one of the G bases from the external G4 layer substituted the missing G base by bending to the free position. Interestingly, the substitution of the G base from the peripheral layer did not imply the disruption of the external layer. Finally, the authors emphasized the fundamental role of the central cations in the stability of the GQ structure, which was verified by additional molecular dynamics simulations with constrained K^+^ ions.

## Figures and Tables

**Table 1:** The special characteristics of analytical methods used in the identification of oxidative lesion of guanine in nucleic acid derivatives.

Method(s) Used ^1^	Compound Detected	Specificity and Other Characteristic(s)	Reference
CV, DPV	o^8^dGuo	uricase treatment eliminated UA interference	Hao et al., 2018 [26]
EIS	o^8^dGuo	the UA peak potential was different (+0.18 V) and of lower intensity than that of o^8^dGuo (+0.37 V)	Manavalan et al., 2018 [27]
EIS, CV	o^8^dGuo	well-separated oxidation peaks for o^8^dGuo, UA and DA	Guo et al., 2016 [28]
LSV	o^8^dGuo	temperature-dependent, kinetic and thermodynamic parameters have been obtained as well	Shang et al., 2018 [29]
SWV	DNA	a single oxidized nucleobase in 6000 can be detected in thin metallopolymer films	Mugweru et al., 2004 [30]
ECL	DNA	thin metallopolymer films can detect OG lesion in DNA	Dennany et al., 2004 [31]
DPV	o^8^dGuo	uricase treatment eliminated UA interference	Cao et al., 2019 [32]
EIS, CV, SWV	o^8^dGuo	o^8^dGuo–aptamer–DNAzyme complex	Zheng et al., 2019 [33]
aptasensor, EIS, DPV	DNA	HCR-enhanced detection of oxidative lesion using GQ formation	Jia et al., 2018 [34]
ECL	o^8^dGuo	multiple-mechanism-driven sensor based on GQ/hemin aptamer; extreme sensitivity	Lv et al., 2018 [35]
fluorometric immunoassay	DNA	it requires o^8^dGuo isolated from damaged DNA	Hu et al., 2019 [36]
fluorometry	o^8^dGuo	AuNP-attached 3D DNA nanomachine	Wei et al., 2018 [37]
colorimetry, CD	o^8^dGuo	naked eye detection of o^8^dGuo–GQ-aptamer	Wang et al., 2014 [38]
luminescence, colorimetry	o^8^dGuo	naked eye detection of o^8^dGuo–GQ-aptamer	Ammanath et al., 2018 [39]
RLS	o^8^dGuo	o^8^dGuo–GQ-aptamer, MNPs	Tao et al., 2018 [40]
nanosecond time-resolved UV spectroscopy	DNA GQ	transient guanine radicals appear as precursors to oxidative damage, generated by absorption of UV radiation	Banyasz et al., 2017 [41]
GC–MS	OG	moderate diagnostic performance	Rozalski et al., 2015 [43]
EC–LC–MS	G, o^8^(d)Guo, short DNA	inter-strand and intra-strand cross-links have been observed beyond oxidation upon EC interaction	Oberacher et al., 2015 [44]
MSPE, HPLC–MS	o^8^dGuo	aptamer-based sample concentration using magnetic forces; enhanced sensitivity	Gan et al., 2018 [45]

^1^ The underlined methods are used for quantitation.

## Abbreviations

2Ih	5-carboxamido-5-formamido-2-iminohydantoin
α-HL	α-hemolysin
AuNP	gold nanoparticles
bpy	2:2’-bipyridine
C	cytosine
c^7^G	7-deazaguanine
CD	circular dichroism
CNNS	carbon nitride nanosheets
CQD	carbon quantum dot
CV	cyclic voltammetry
DA	dopamine
dG	2’-deoxyguanosine in ON sequences (the prefix d in front of parenthesis relates to the whole sequence)
DMAc	*N,N*-dimethylacetamide
DPV	differential pulse voltammetry
(d)Rib*f*	(2-deoxy-)b-d-ribofuranosyl
dsDNA	double-stranded DNA
DW	DNAwalker
EC-LC-MS	electrochemistry-liquid chromatography-mass spectrometry
ECD	electrochemical detection
ECL	electrochemiluminescence
EIS	electrochemical impedance spectroscopy
Fapy-G	2,6-diamino-4-hydroxy-5-formamidopyrimidine
G	guanine (in ON sequences guanosine)
G4	guanine tetrad
GCE	glassy carbon electrode
Gh	5-guanidinohydantoin
Gh^ox^	5-guanidinodehydrohydantoin
GN	graphite nanosheets
GO	graphene oxide
GQ	guanine quadruplex
HCR	hybridization chain reaction
hm^5^Ura	5-hydroxymethyluracil
HPLC	high-performance liquid chromatography
hTel	human telomere
Iz	2,5-diaminoimidazolone
LOD	limit of detection
LSV	linear sweep voltammetry
m^5^C	5-methylcytosine
MB	methylene blue
MCH	6-mercapto-1-hexanol
MNP	magnetic nanoparticles
MWCNT	multi-walled carbon nanotubes
NEase	nicking endonuclease
NEIL3	Nei-like or endonuclease VIII-like 3 enzyme
NHE	normal hydrogen electrode
NMR	nuclear magnetic resonance
NOE	nuclear Overhauser effect
nt	nucleotide
o^8^dGuo	8-oxo-7,8-dihydro-2′-deoxyguanosine
OG	8-oxo-7,8-dihydroguanine
OGG1	OG-glycosylase 1
ON	oligonucleotide
PSWCNT	porous single-walled carbon nanotube
PT	poly(3-alkoxy-4-methylthiophene)
PVP	poly(vinylpyridines)
QCM	quartz crystal microbalance
rGO	reduced graphene oxide
RAD17	the gene encoding cell cycle checkpoint protein RAD17
RLS	resonance light scattering
RNS	reactive nitrogen species
ROS	reactive oxygen species
SEM	scanning electron microscopy
S/N	signal-to-noise ratio
SOD	superoxide dismutase
Sp	spiroiminodihydantoin
SPCE	screen-printed carbon electrode
ssDNA	single-stranded DNA
SWCNT	single-walled carbon nanotube
SWV	square wave voltammetry
TD-DFT	time-dependent density-functional theory
TEM	transmission electron microscopy
TGA	thermogravimetric analysis
UA	uric acid
XRD	X-ray diffractometry
Z	2,2,4-triamino-2*H*-oxazol-5-one
